# Supplementary figures and images for: A nanocompartment system contributes to defense against oxidative stress in Mycobacterium tuberculosis
Source: eLife. 2021 Nov 9;10:e74358. doi: 10.7554/eLife.74358 (PMC8635971; doi:10.7554/eLife.74358)

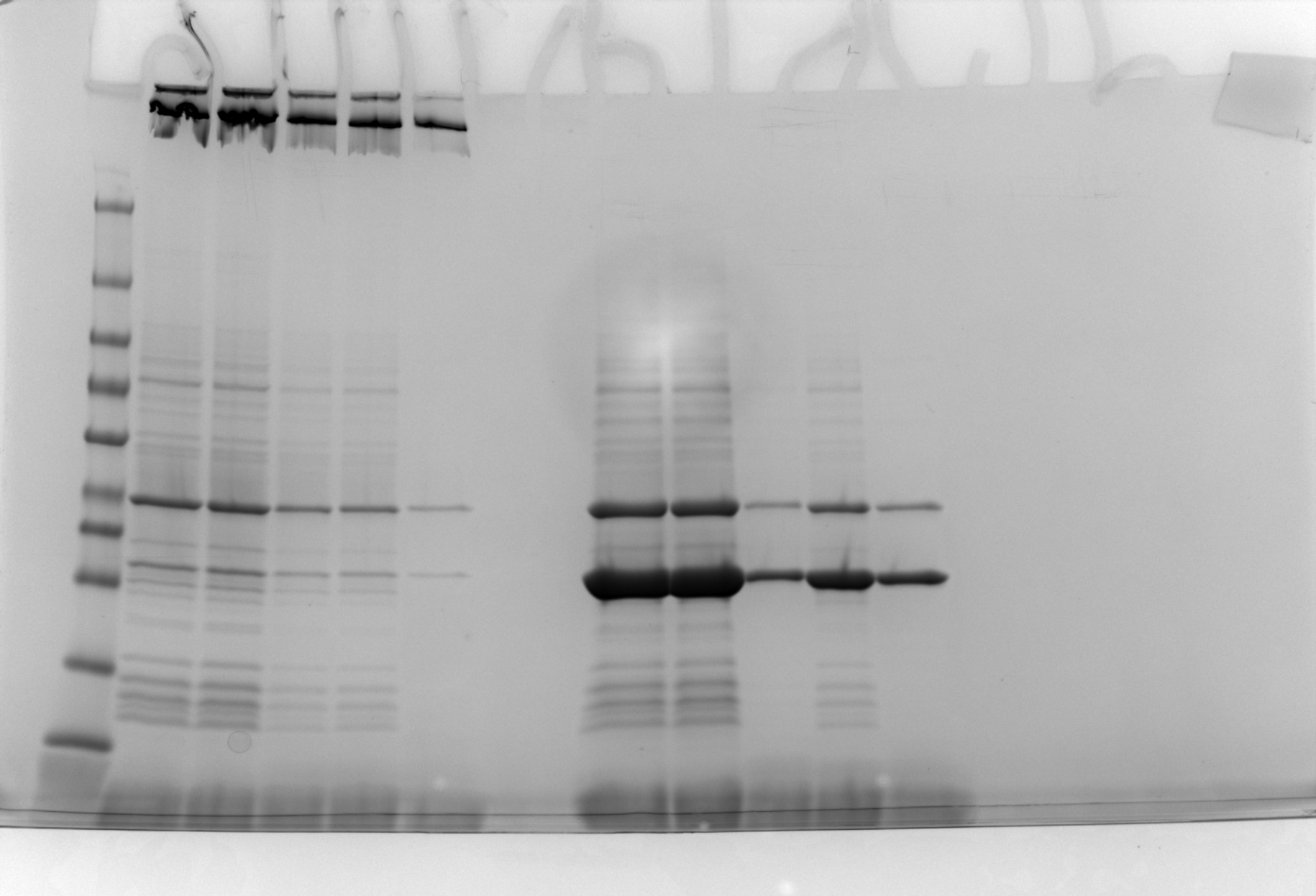

Supplement: Source data 1. [file elife-74358-supp2.zip › Source Data File 1.tif]

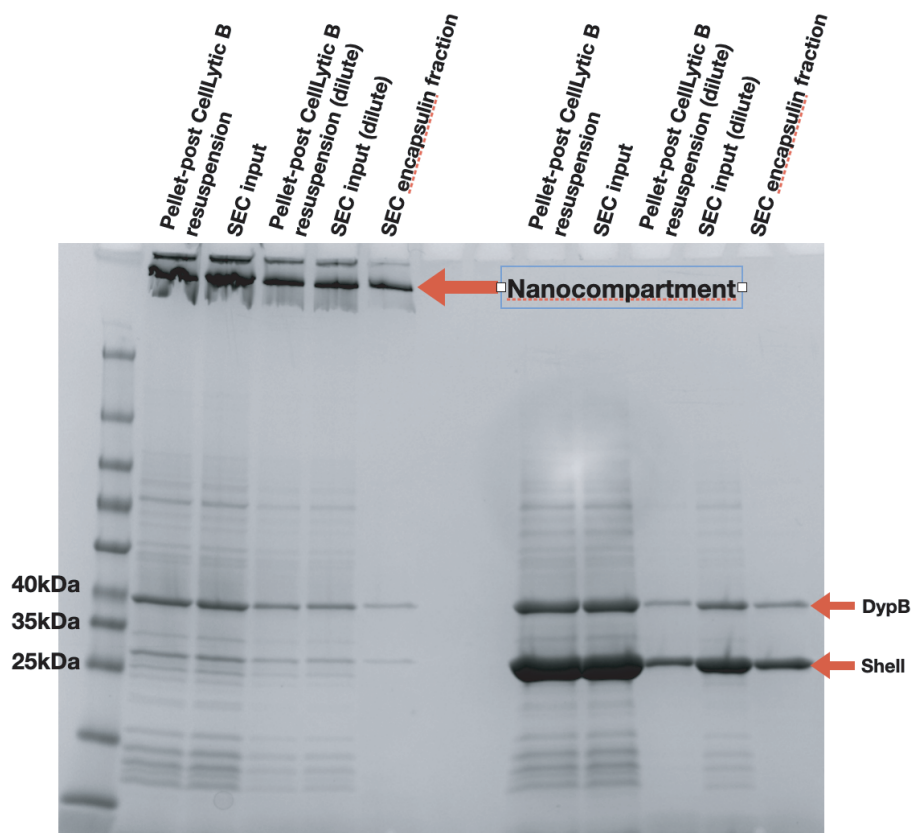

Supplement: Source data 2. [file elife-74358-supp3.zip › Source Data File 2.pdf]

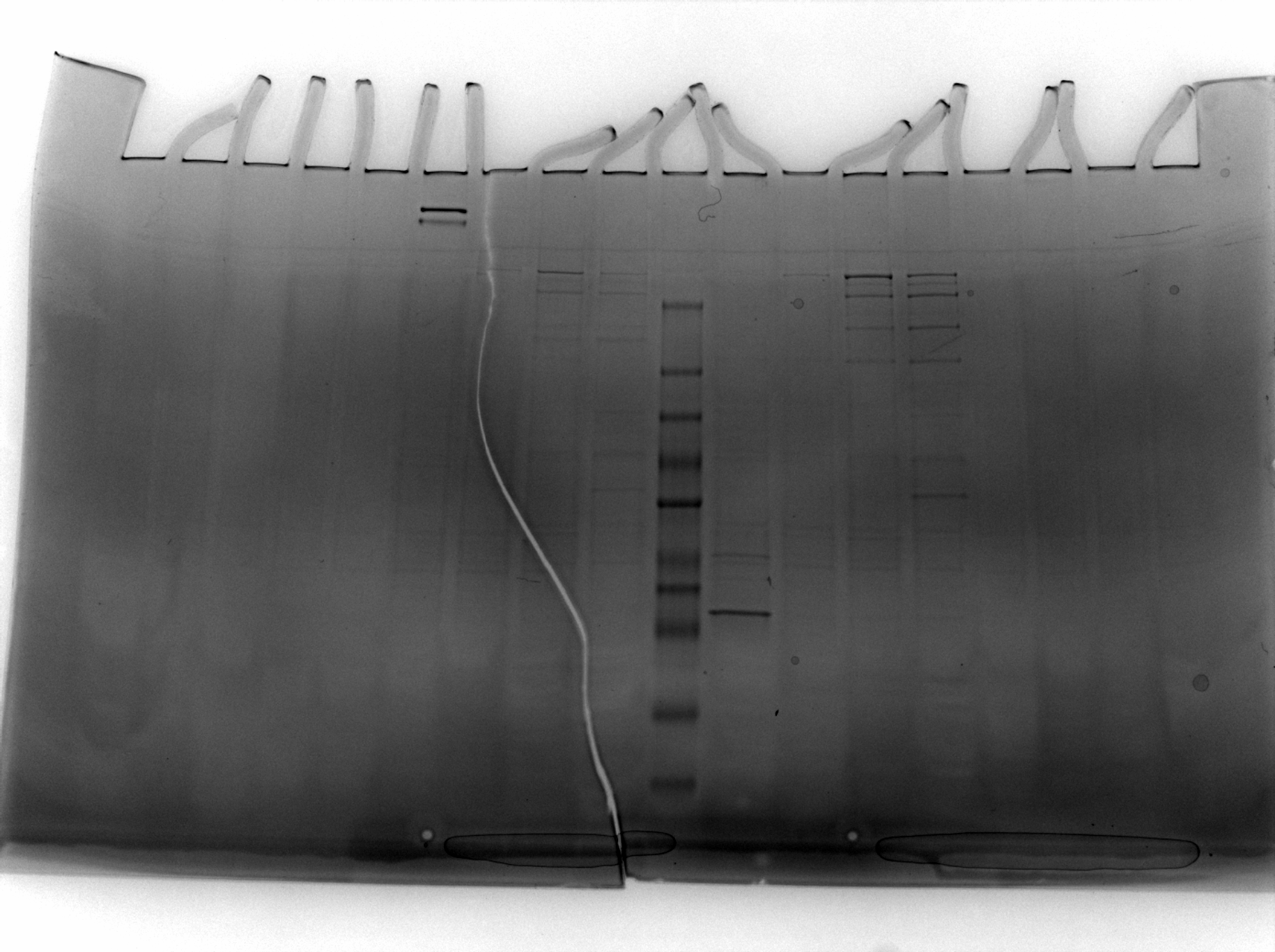

Supplement: Source data 3. [file elife-74358-supp4.zip › Source Data File 3.tif]

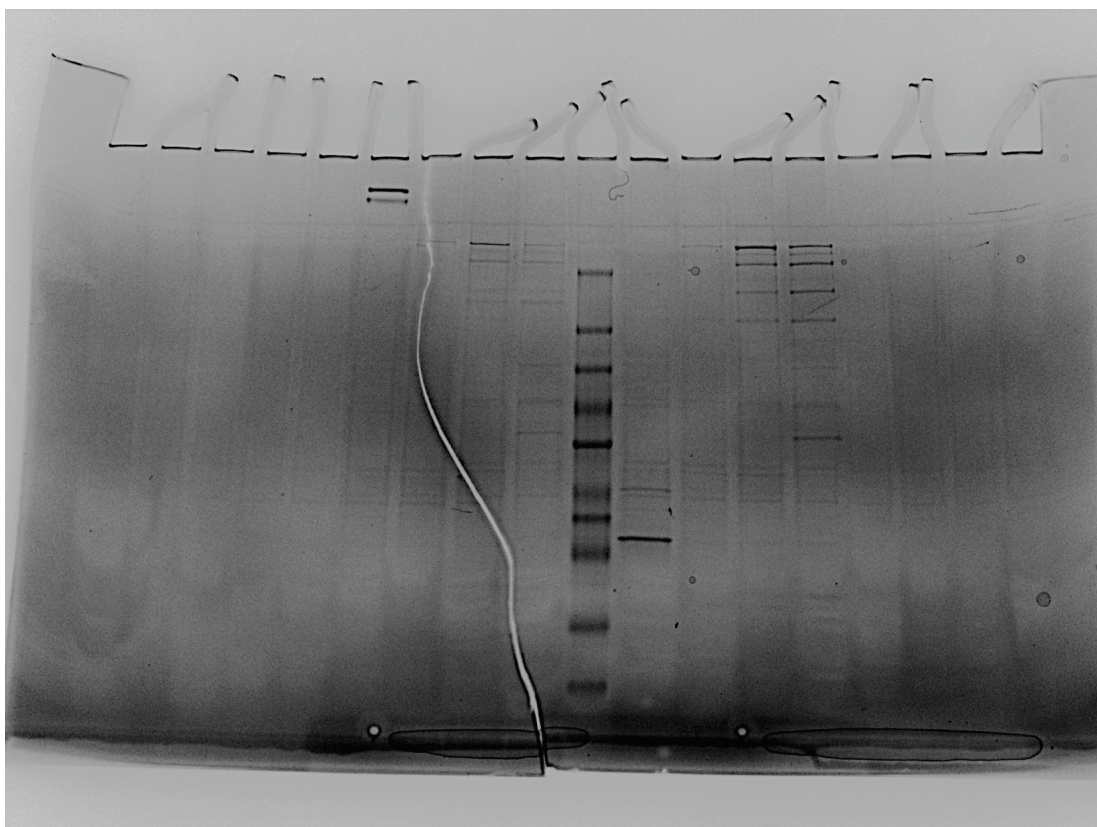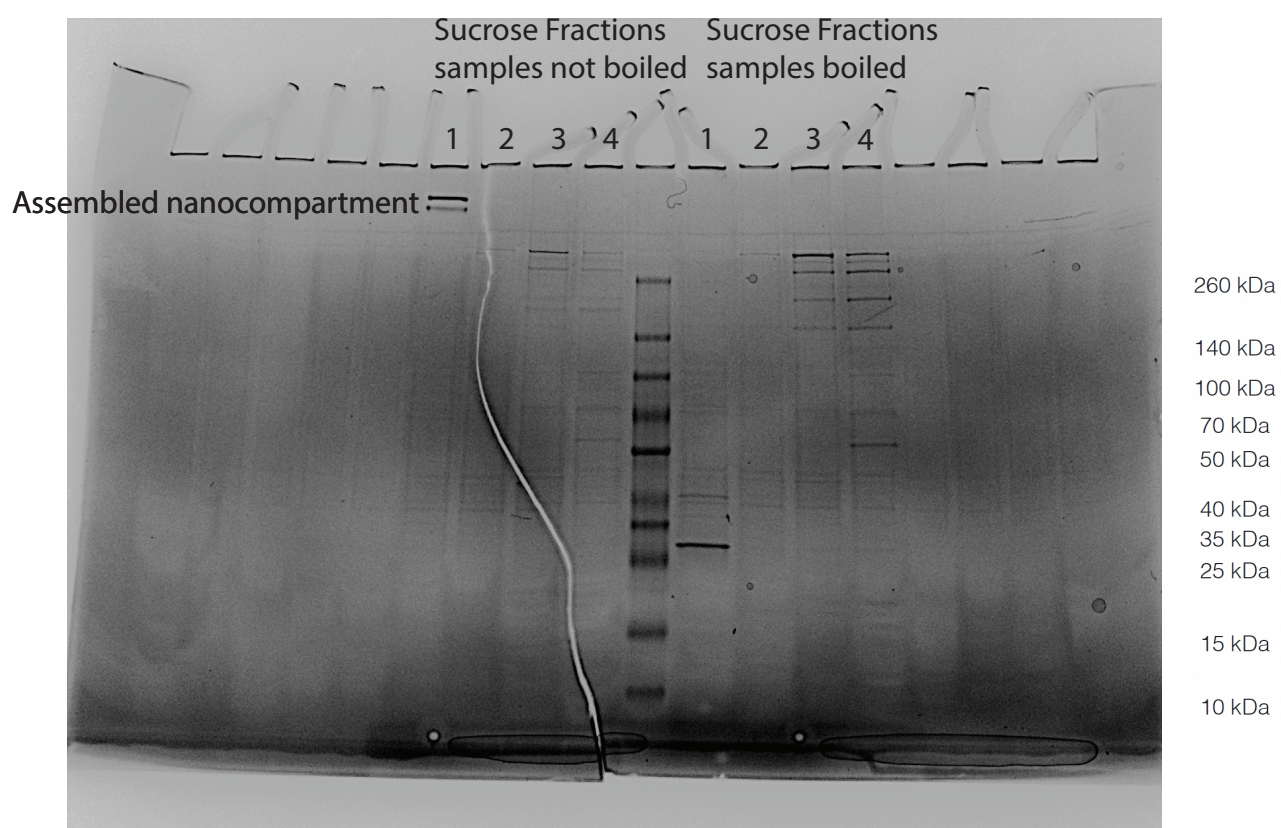

Supplement: Source data 4. [file elife-74358-supp5.zip › Source Data File 4.pdf]

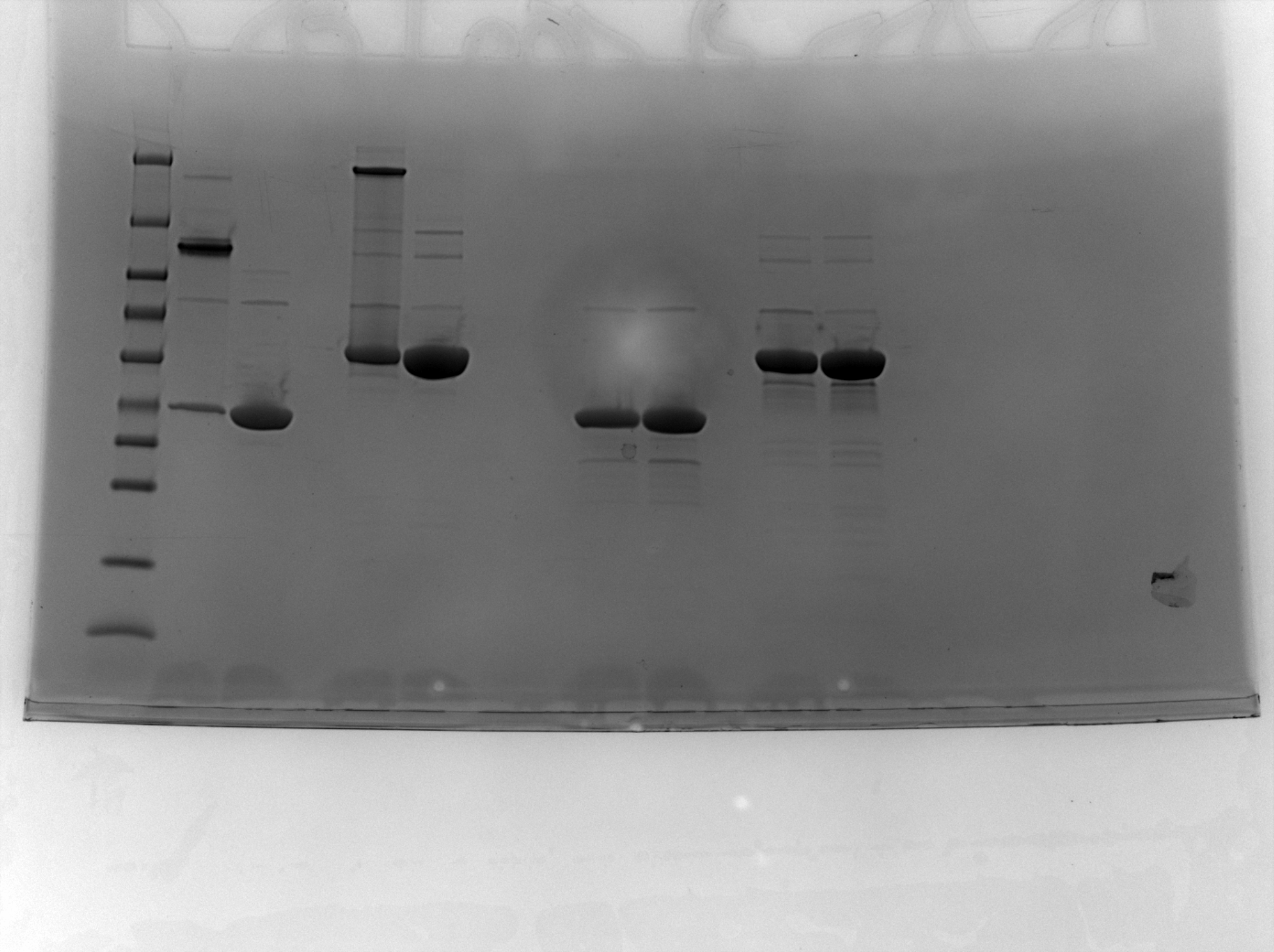

Supplement: Source data 5. [file elife-74358-supp6.zip › Source Data File 5.tif]

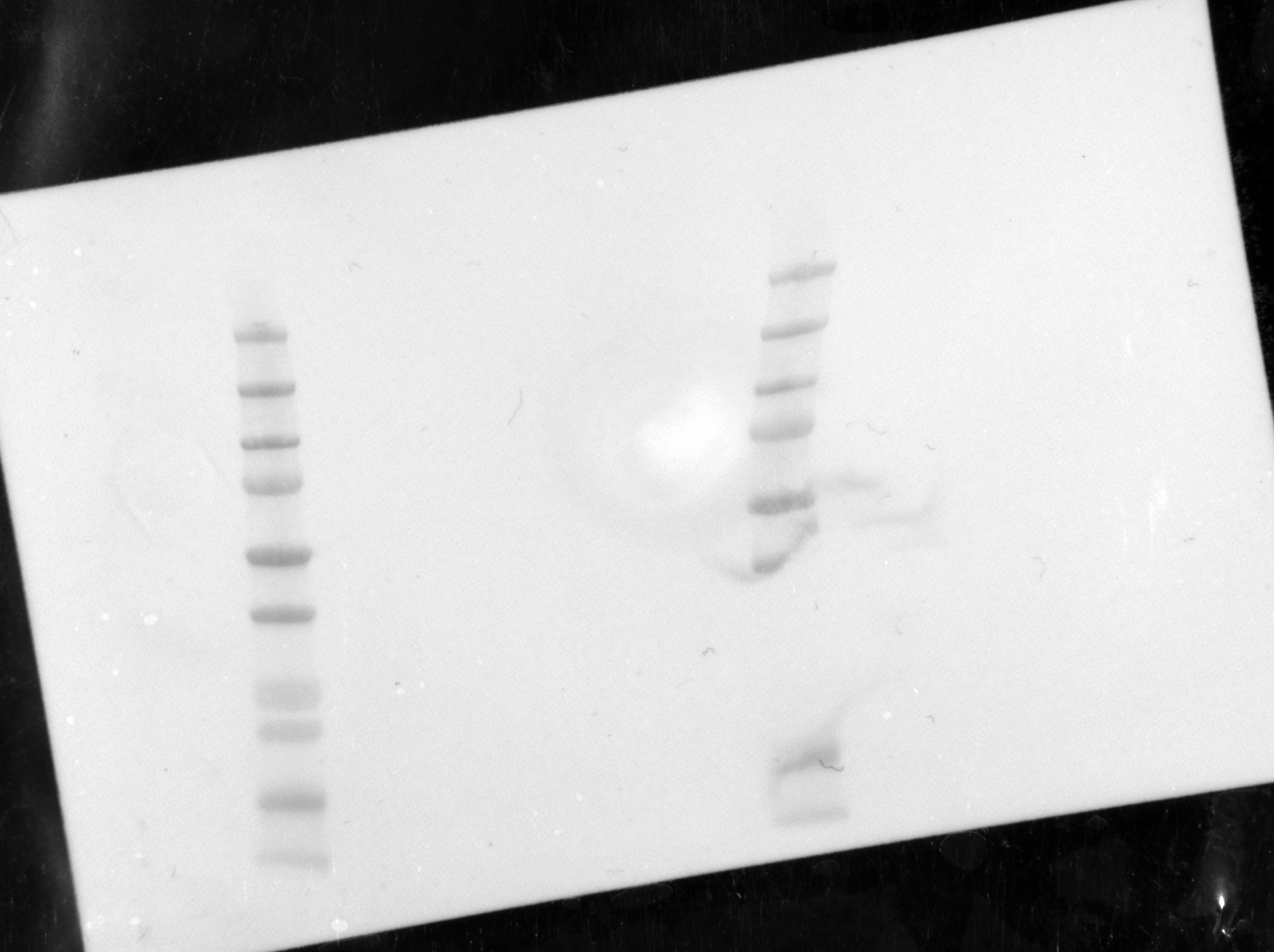

Supplement: Source data 6. [file elife-74358-supp7.zip › Source Data File 6.tif]

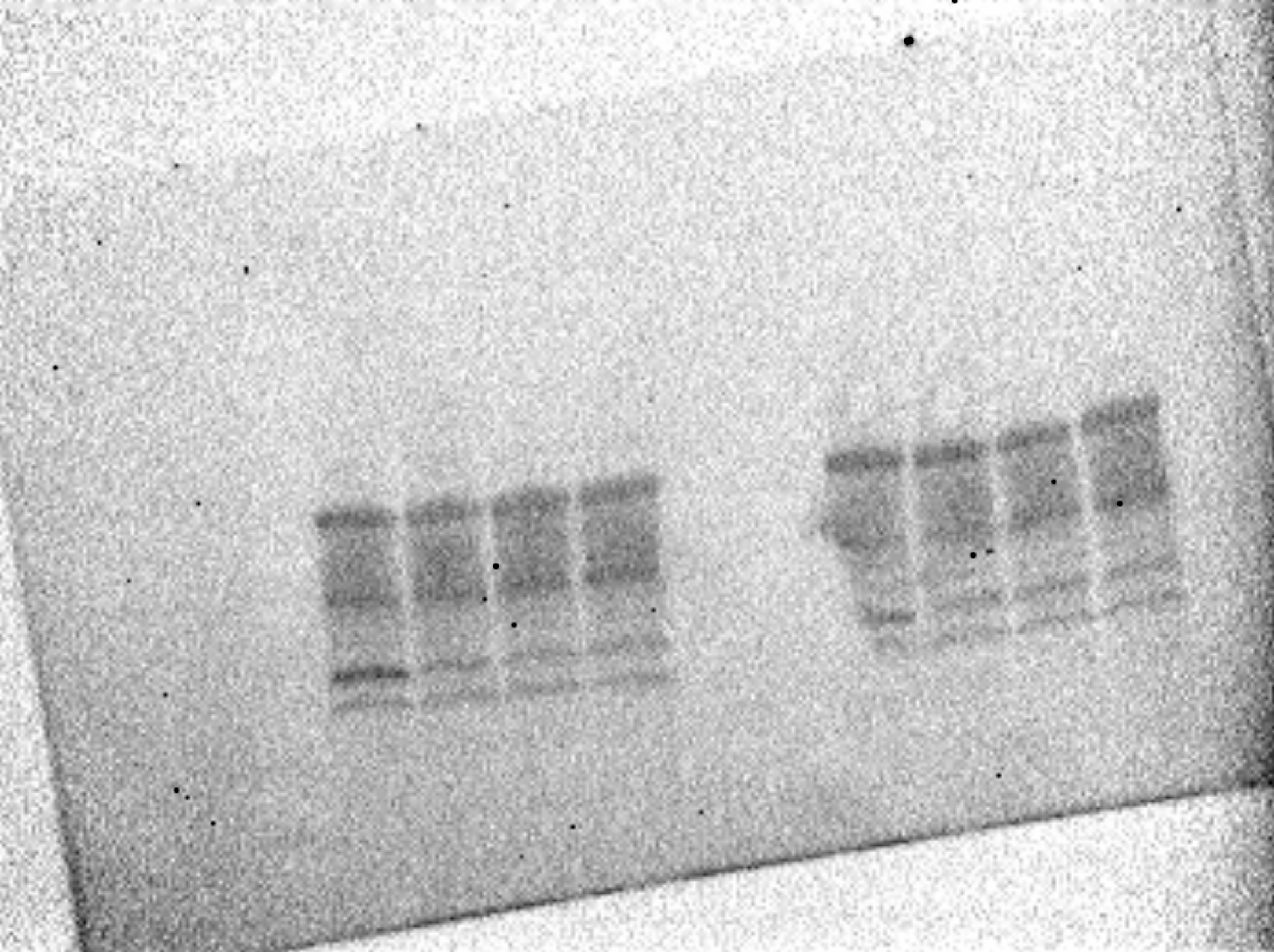

Supplement: Source data 7. [file elife-74358-supp8.zip › Source Data File 7.tif]

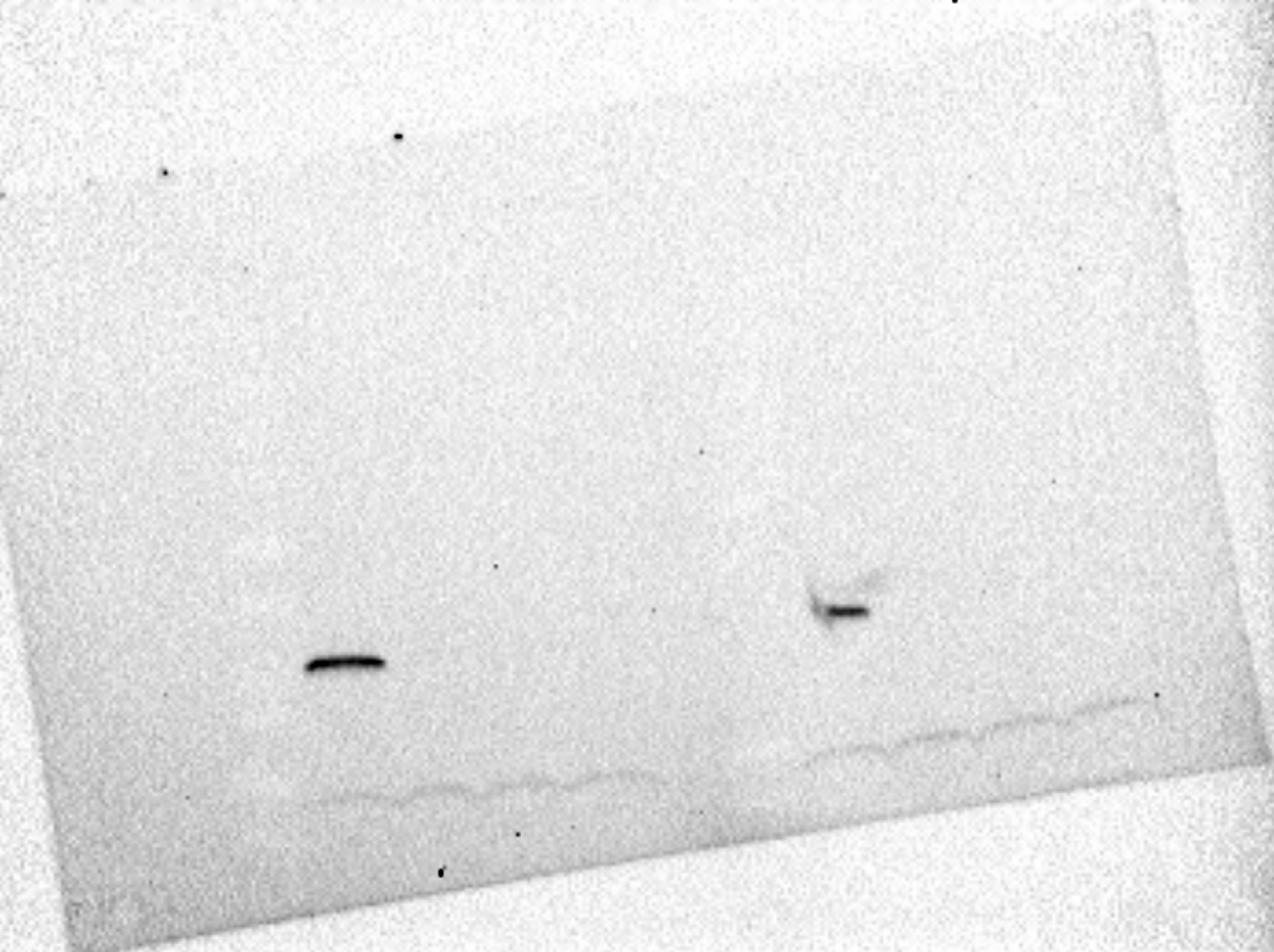

Supplement: Source data 8. [file elife-74358-supp9.zip › Source Data File 8.tif]

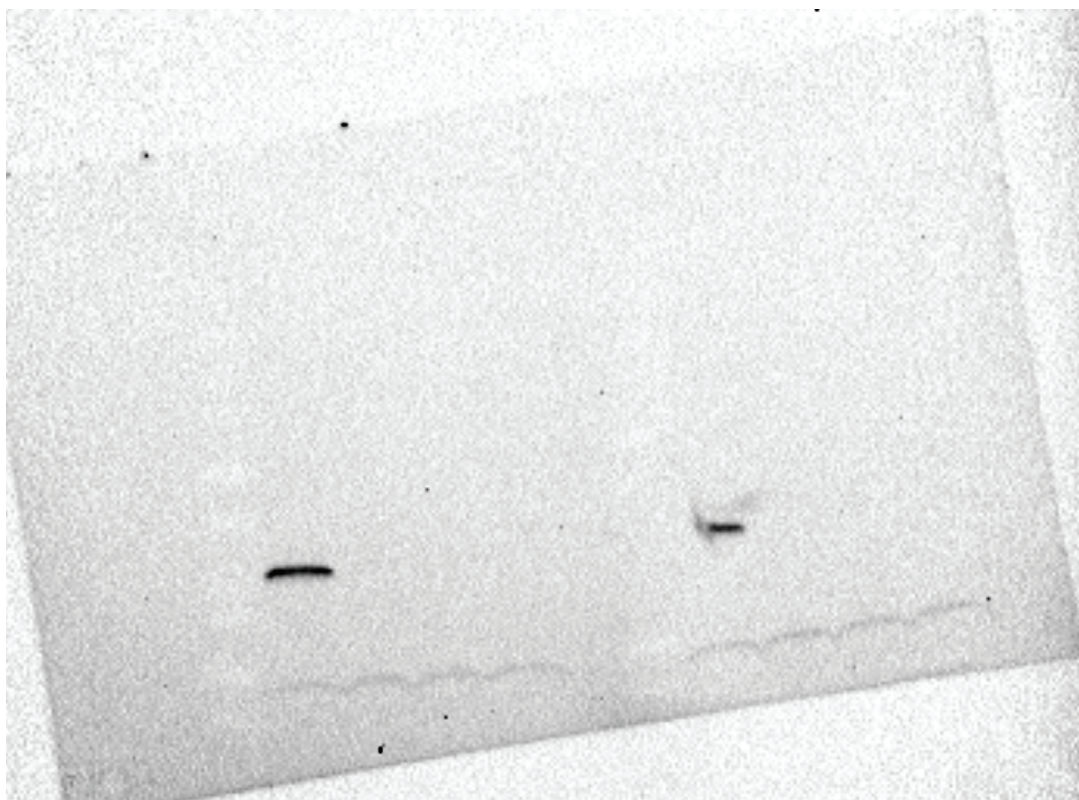

Rv Op 98 99

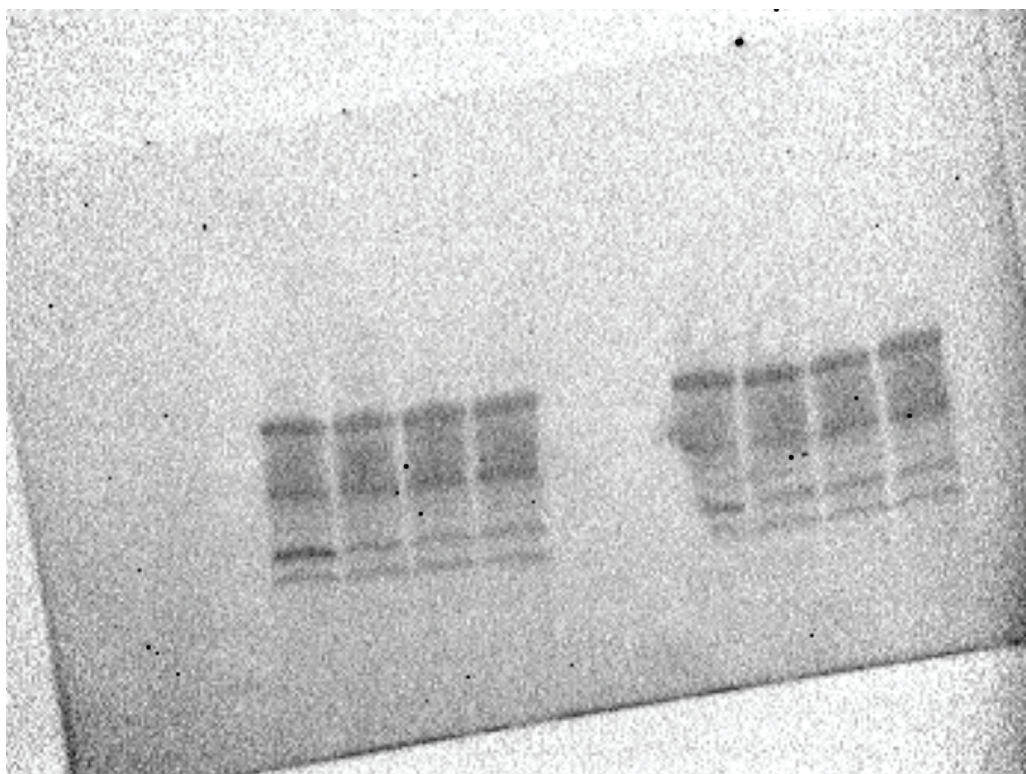

Rv Op 98 99

Supplement: Source data 9. [file elife-74358-supp10.zip › Source Data File 9.pdf]

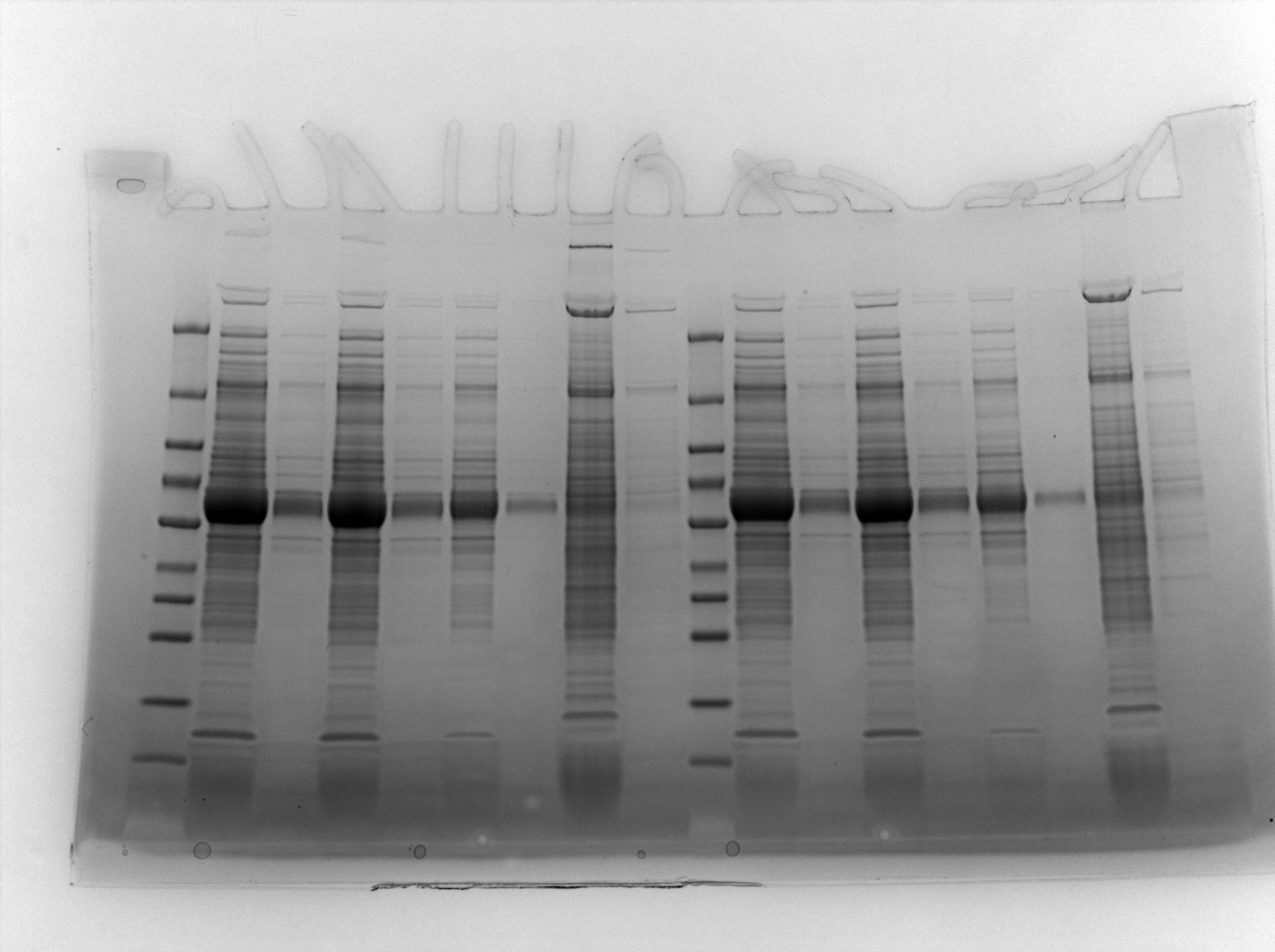

Supplement: Source data 10. [file elife-74358-supp11.zip › Source Data File 10.tif]

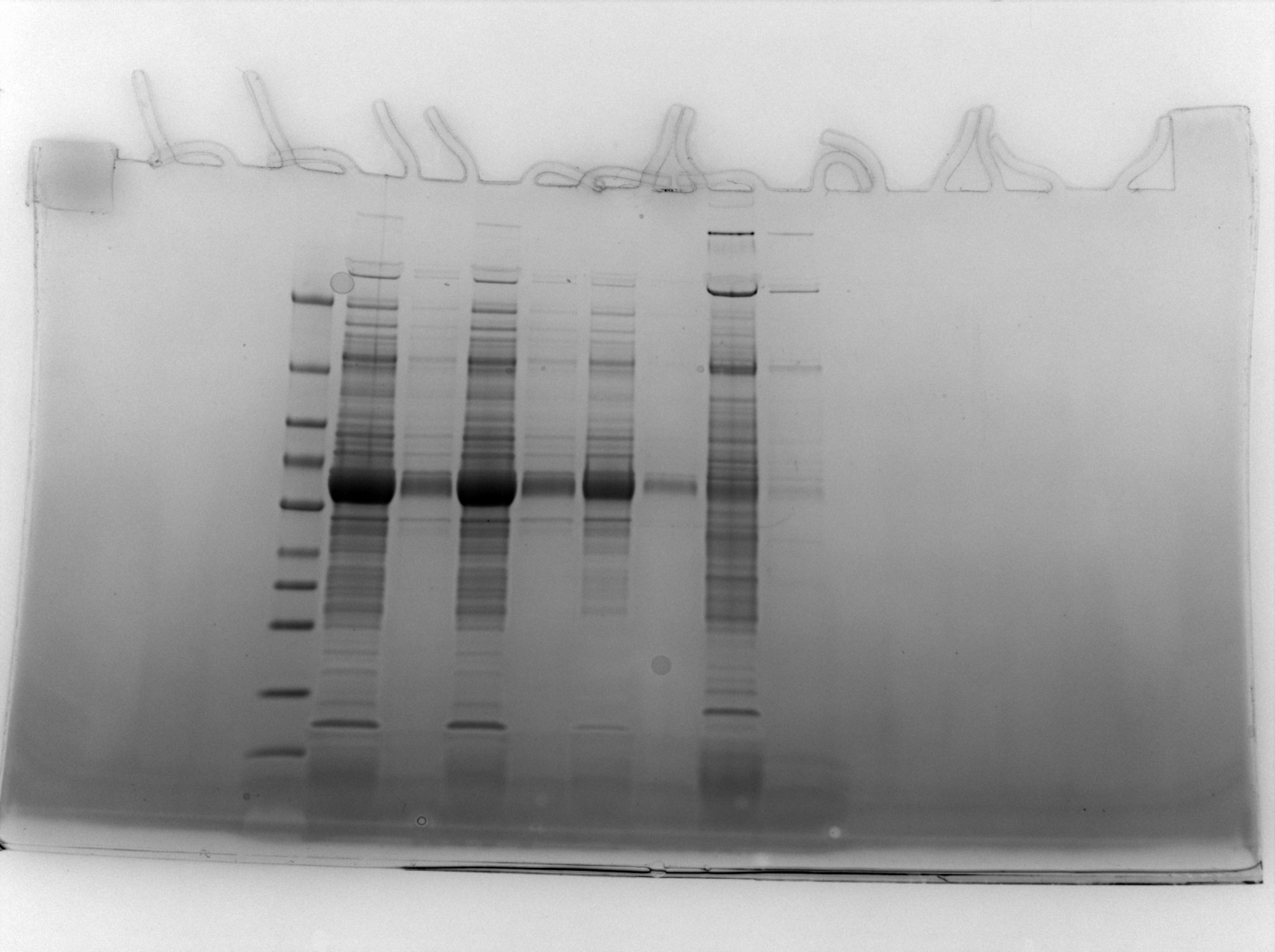

Supplement: Source data 11. [file elife-74358-supp12.zip › Source Data File 11.tif]

### Tn::DypB+DypB

### Tn::DypB+Cfp29

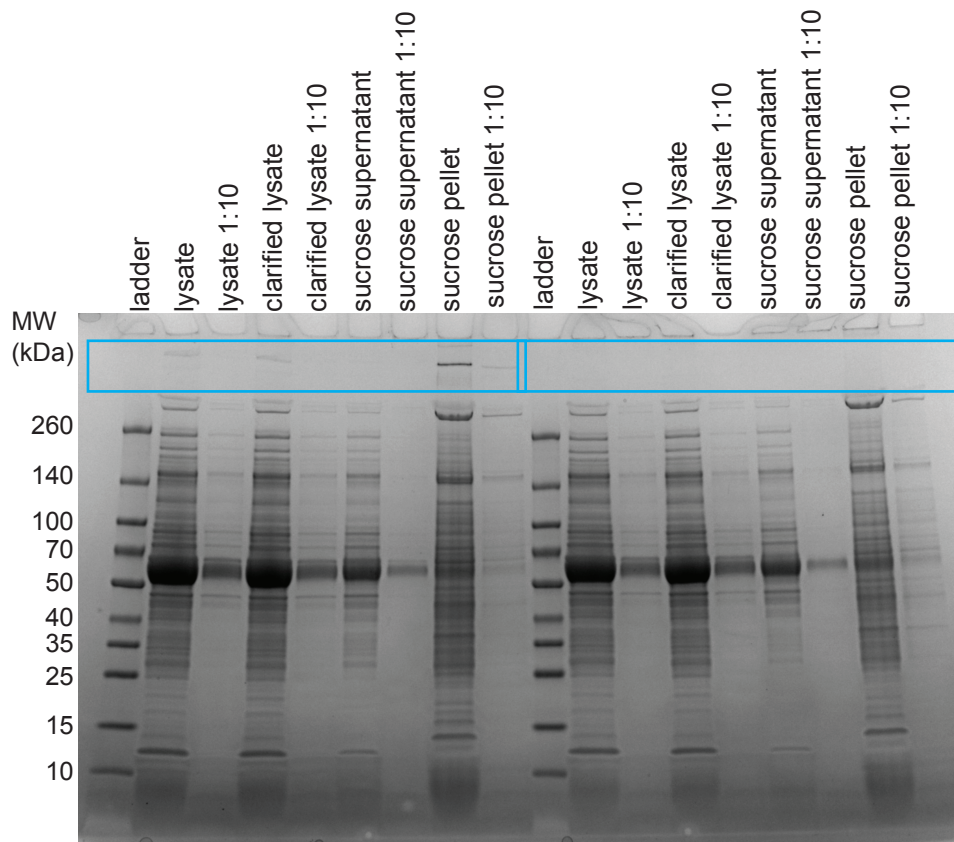

### Tn::DypB+operon

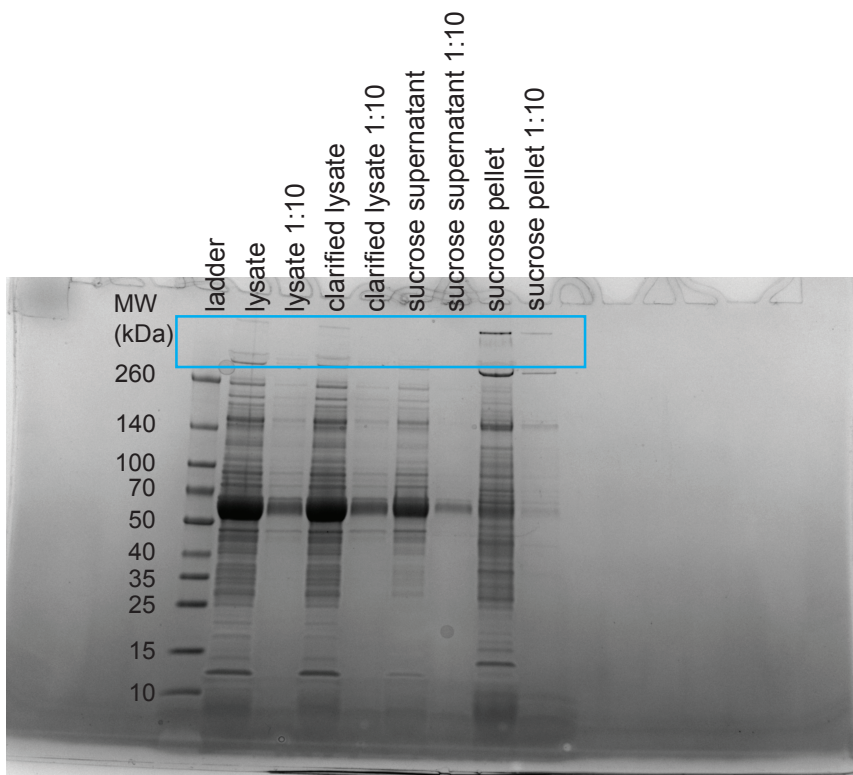

Supplement: Source data 12. [file elife-74358-supp13.zip › Source Data File 12.pdf]
